# Investigation of Natural Dyes and Taxonomic Identification of Fibers Used in Chancay Textiles by Vibrational Spectroscopy and Mass Spectrometry

**DOI:** 10.1021/acs.jproteome.4c00809

**Published:** 2025-01-03

**Authors:** Katja S. Diaz-Granados, Laura J. Bergemann, Mary Ballard, G. Asher Newsome, Gwénaëlle M. Kavich, Joshua D. Caldwell, Timothy P. Cleland

**Affiliations:** † Interdisciplinary Materials Science, Vanderbilt University, Nashville, Tennessee 37212, United States; ‡ The Conservation Center, Institute of Fine Arts, New York University, New York, New York 10075, United States; § Museum Conservation Institute, Smithsonian Institution, Suitland, Maryland 20746, United States; ∥ Department of Mechanical Engineering, Vanderbilt University, Nashville, Tennessee 37212, United States

**Keywords:** Archaeological textiles, Natural dyes, Animal fibers, Keratin, Chancay, LC-MS, DART-MS, ATR-FTIR

## Abstract

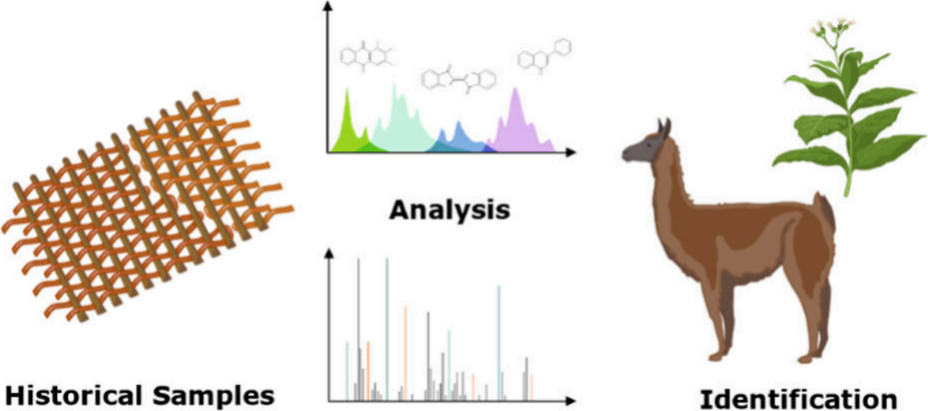

Textiles provide a valuable source of information regarding past cultures and their artistic practices. Understanding ancient textiles requires identifying the raw materials used, since the origin of dyes and fibers may be from plants or animals, with the specific species used varying based on geography, trade routes and cultural significance. A selection of nine Chancay textile fragments attributed to 800–1200 CE were studied with liquid chromatography mass spectrometry (LC-MS) and direct analysis in real time mass spectrometry (DART-MS) to identify the chemical compounds in extracts of natural dyes used to create green, blue, red, yellow and black colors. From the identified molecular markers, the green colors involved the overdyeing of indigo and flavonoid dyes, the blue colors were achieved using an indigo dye, the yellows came from a flavonoid dye, the reds from anthraquinone dyes of both plant and animal origin, and the black from a mixture of flavonoid, anthraquinone and indigo dyes. A subset of the textiles was identified as containing proteinaceous fibers based on ATR-FTIR. These textiles were further studied using a mass spectrometry-based proteomics approach to identify the species used, with the peptide sequences measured confirming the presence of South American camelids, most likely llama or alpaca.

## Introduction

The Chancay were a civilization living in coastal Peru, where they exercised control over the Chillon, Huaura, Rimax, Lurin and Chancay valleys, during the Late Intermediate Period (A.D. 1000–1476).^1^ Textiles were incredibly important to pre-Columbian Andean societies, with elaborately woven fabrics serving as symbols of social status and political importance. During the Late Intermediate Period, textiles both were a highly valued commodity and had reached advanced levels of artistry. The well-developed culture of weaving and textile manufacturing included a wide palette of dyes used to achieve intricate patterns, often with highly complex symbolic meaning,^2^ and the chain of production for textiles employed a large fraction of the populace, with labor required to cultivate and collect the raw material for fibers, card and spin the fibers into threads and then dye and weave the final piece. Chancay weavers are credited with multiple technological innovations, including the development of a knotted weft wrapping technique which was often paired with embroidery to create sophisticated patterned textiles.^3^ Fortunately, many of the textiles from this time frame have survived thanks to the cold and arid conditions of burial sites in this high-altitude region.^4^ Indeed, the favorable conditions for preservation mean that Chancay textiles are the most abundant record of ancient textiles that survive, forming the largest continuous record of textiles that we have for any group of civilizations.^5,6^

The practice of coloring textiles using dyes began as early as 4000 years ago, and until the advent of synthetic dye manufacture in the later part of the 19th century, relied entirely on plant and animal dye sources. An understanding of the types of dyes used in ancient artifacts can inform researchers on when an object was created, the types of technologies and trade routes available at the time, and what considerations to take into account when storing, treating and exhibiting fragile dyed textiles. Similarly, the choice of species used to create the textile fibers can reflect the natural resources available, as well as the cultural and political environment in which the objects were made. For instance, in ancient Peru, fibers were generally prepared from either cotton or animal hair, with some preference for cotton fibers in coastal areas and for animal hair in the higher altitude grassland regions.^7^

In the absence of written records, our present understanding of the dyeing and fiber technology of the Chancay civilization is inferred entirely from analytical techniques. Different analytical techniques will require different amounts of material, but, as with all cultural heritage objects, there are limitations imposed on the quantity of textile fibers that can be extracted for analysis. Many of the standard methods for dye and protein identification involve destructive chemical tests, which prohibit a single sample from being stored for future use. Even when sufficient material can be measured, dye identification is complicated by multiple factors, including that (1) little information remains regarding the original recipes used to prepare natural dyes, (2) the color resulting from natural dyes is often due to the coexistence of multiple chromophores and associated compounds, (3) the high molar extinction coefficients of organic compounds and their corresponding high tinting strength means that small concentrations of dyes were often used to achieve bold colors and (4) the degradation of the original materials can cause the appearance of secondary products, as well as distorting the visual appearance of the intended color.^8^ Similarly, species identification for protein-based fibers is frustrated by (1) the low solubility of cross-linked proteins, (2) the limited number of species for which sequencing data is available in protein databases and (3) the potential degradation and chemical modification to proteins due to aging. Natural dye identification and fiber species identification are often treated separately, but for the purposes of cultural heritage research, both provide relevant information about the nature of the material. Given the limitations on sampling from cultural heritage objects, ideal strategies for both identifications should be complementary, either due to their nondestructive nature, or by allowing a simplified chemical extraction to be used for both dye and protein retrieval.

In this work, a multianalytical approach, involving Attenuated Total Reflectance-Fourier Transform Infrared (ATR-FTIR) spectroscopy and mass spectrometry, was taken for the comprehensive identification of both the dyes and fibers used in a selection of Chancay textiles. Two mass spectrometry techniques were employed. The first was direct analysis in real time mass spectrometry (DART-MS), which relies on thermal desorption and ionization using a helium postplasma gas stream. The second was liquid chromatography mass spectrometry (LC-MS). The DART-MS technique proved to be an efficient method for characterizing general classes, since individual scans could be run in under a minute, with little to no sample preparation. Although DART-MS data were less comprehensive, especially for dye mixtures containing isomers, the speed and sensitivity of the measurements meant that rapid scans could be taken for preliminary screening to determine where further analysis was needed. LC-MS allowed for a more detailed review of both the chromophores present in the dyes and the peptide sequences of the wool-based fibers. The focus of the work was to better understand the relationship between primary and secondary dye colors and the choice of animal species used to create textiles, as well as to develop a workflow to accommodate the joint analysis of dye composition and fiber type.

## Natural Dye Identification

Like all materials, natural dyes are subject to varying degrees of degradation, which occur due to contact with other materials, as well as exposure to light, humidity and oxygen. The dyes in ancient samples will have undergone even more complex degradation processes due to their exposure to microbiological agents and soil.^9^ After being buried for long periods of time, textiles will have lost much of their color due to leaching processes involving exposure to groundwater, and if they have been buried near metal objects or in soils with high metal contents, they may also have undergone mineralization processes spurred by the metal’s corrosion.^10^ The fact that dyes are so sensitive to damage stresses the importance of understanding the chemical makeup of organic dyes, in order to halt their decay as much as possible through appropriate exhibition and storage conditions and to avoid restoration treatment that could further aggravate the loss of their colors. Because the chemical makeup of dyes can involve mixtures of chromophores, however, many of which have multiple potential sources,^11^ identifying the materials used to color textiles can be particularly challenging.

Most early work on natural dye identification was done using microchemical analysis, wherein different dyes would be made to produce diagnostic colors upon the application of specific solvents,^2^ or using optical techniques, like UV–visible (UV–vis) spectroscopy. UV–vis spectroscopy in particular was a standard method, as it could characterize organic dyes based on the electronic transitions of dyeing molecules, offering a straightforward means of separating general classes of materials. The downside was the strong tendency toward overlap between the relatively broad bands characteristic of organic colorants, which was exacerbated by the surface texture of textiles.

To address the complexity of natural dyes, which are often composed of multiple colored and uncolored compounds, a separation technique can be used to isolate each compound prior to detection. Natural dyes are organic compounds and thus can be separated by passage through a liquid chromatography column, particularly with reverse phase gradients. A significant improvement in identification capabilities was reached in the 1980s by pairing chromatographic separation (e.g., high-performance liquid chromatography) with the high sensitivity of UV–vis detectors^12^ or diode array detectors (DAD).^13,14^ More recently, LC-DAD has been used to identify natural dye in 4–12th century Egyptian textiles,^15^ red dyes in ancient Peruvian textiles,^16^ yellow dyes in Andean textiles^17^ and red and blue dyes in Andean textiles.^18^

Pairing liquid chromatography with spectroscopic detection provided improved identification confidence, allowing researchers to match dyes on the basis of both their measured retention times and their absorbance spectra. Currently, the techniques used for natural dye identification have expanded to include hyphenated techniques that match liquid chromatography with mass spectrometry. Mass spectrometry can complement the color information provided by spectroscopic detection techniques in multiple ways. To begin with, many of the compounds that produce similar absorption spectra can be differentiated on the basis of their molecular mass. LC-MS makes it possible to identify auxiliary colorless compounds, which may not be directly responsible for the observed color of the textile, but which nevertheless may hold valuable information about the composition of the dye mixture. The pattern of mass peaks can also be used to infer structural information, which becomes particularly important when attempting to reconstruct unknown chemical compounds, especially given the limited available references for natural dyes. Additionally, a comparison of diode-array UV–vis and MS found the limit of detection to be lower for most compounds with MS detection^19^ and with MS it is possible to avoid some of the matrix interference effects that come from wool degradation products and may make UV–vis data difficult to interpret.

By pairing liquid chromatography with mass spectrometry, the assignment of dye compounds is based on multiple data points, including retention time through the LC column, the measured molecular mass and possibly the masses of product ions (if operating in tandem MS mode). This allows for coeluting compounds to be distinguished based on their masses (and potentially their fragmentation patterns). The opportunity for tandem mass spectrometry has made it possible to collect structural information useful for characterizing unknown chemical compounds (e.g., understanding the glycosylation of chromophores in yellow flavonoid dyes^11^). Likewise, isomers with the same empirical formula, which cannot be differentiated through their mass spectra alone, can be compared on the basis of retention time. Having multiple independent means of identification has helped remove ambiguity when studying dyes with similar chemical compositions. LC-MS has been shown to work well for characterizing complex extracts containing mixtures of flavonoids from plant material, either in the context of dyestuffs or food extracts,^20,21^ and various groups have conducted detailed investigations into the exact fragmentation mechanisms and expected observed ions for flavonoids measured under different ionization conditions.^22,23^ LC-MS has also been applied to study natural dyes in historical artifacts, including alizarin by Yamaoka et al.,^24^ flavonoid yellows by Ferreira et al.^25^ and natural Chinese dyes by Liu et al.^26^

In general, the low analyte concentration of natural dyes and the potential use of mordants mean that an extraction step is needed to isolate the dyestuff prior to characterization with LC-MS. The traditional extraction protocols involve the application of a strong acid like hydrochloric acid and heat, to disrupt the complexation between phenolic groups of the dye chromophore and the metal ions of the mordant.^12^ Unfortunately, many of the established extraction protocols are accompanied by significant chemical alteration of the dye chromophore, including esterification of phenol carboxylic compounds, decarboxylation and dehydration.^27^ For flavonoids, in particular, solvents like HCl will cause the loss of sugars through glycosidic bond cleavage. This leaves behind only the aglycone, which can suggest a broad class of dye plants but prevents more targeted species identification. Gentler extraction methods, especially those that preserve glycosidic bonds, are increasingly being implemented. The goal of these modified extraction protocols has been to extract sufficient dye material while not chemically altering the dye compounds, and to potentially allow for multiple types of information to be derived from a single extraction. This last point is particularly relevant for the analysis of ancient textiles colored using mixtures of dyes, since the small quantities of available material precludes the use of separate extraction protocols geared toward each class of dye compounds. Modified extraction protocols have included the use of alternative acids like HF, which has been shown to efficiently extract pseudo purpurin and alizarin,^28^ trifluoroacetic acid, which has the benefit of extracting turmeric,^29^ and formic acid with methanol, which works well for wool dyed with flavonoid compounds.^30^ The traditional HCl/methanol extraction is also ineffective for vat dyes, like indigo, for which alternative organic solvents like DMSO, pyridine or DMF are needed.^31−33^

Beyond chromatographic techniques, another commonly employed microdestructive analytical technique is direct mass spectrometry, of which there are again many forms (including matrix-assisted laser desorption/ionization mass spectrometry, time-of-flight secondary ion mass spectrometry and direct analysis in real-time mass spectrometry). With direct mass spectrometry one can bypass the need for sample pretreatment, so there is less chance of chromophores being lost due to the choice of extraction procedure, and only very small samples are needed. Direct mass-spectrometry is also both fast and sensitive, which makes it an ideal technique for sorting through the massive number of potential chromophores that can be encountered. The main limitations of mass spectrometry are that many dye sources exist as structural isomers (e.g., indigotin and indirubin or alizarin and xanthopurpurin) and that the technique can have difficulty with materials that are mixtures of multiple pure substances.

In this work we made use of direct analysis in real-time mass spectrometry (DART-MS). DART-MS has been extensively used in the context of forensic science applications, including drug detection, ink characterization and illegal trade prevention. The primary motivation for using DART-MS in cultural heritage research is the minimal sample preparation required. In particular, DART-MS can be useful as a screening technique, as the short exposure times are sufficient for down-selecting which samples require more extensive analysis. Small quantities of sample material can be placed in front of the DART source for a less than a minute of exposure, and the technique has shown success for the identification of small molecules, even in samples with multiple compounds.^16,34−36^

## Species Identification

The natural fibers used to create Chancay textiles can be made from either plant material or animal hair. Often these were combined in a single textile, with a plant fiber used as the warp and animal hair as the weft,^37^ or, in the cases of embroidered textiles, a plant fiber for the substrate fabric and animal hair for the decorative elements. Traditionally distinguishing plant fibers from animal hair has been done on the basis of morphological differences visible under light microscopy or through elemental analysis using SEM-EDS.

Examining plant fibers with optical microscopy will show these to have transverse dislocations, convolution, nodes and a general polygonal cross-section, with slight differences appearing due to species specific traits, as well as the conditions of harvest,^17,38^ while animal hairs have characteristic medulla, diameter and scale patterns. Visual examination of animal hair generally relies on the adequate preservation of diagnostic morphological features, like the overlapping scale patterns on the outer cuticle and the diameter of the fiber, both of which may have been modified over long periods of time due to abrasion or microbial decomposition. In extreme cases, the fibers may even have been partially replaced through mineralization, leaving behind a pseudomorph of the original textile. Interpreting the appearance of fibers using microscopy can also be somewhat subjective and relies heavily on both the operator’s expertise and the condition of the fiber, which may be influenced by aging, or the processing involved in dyeing.

Alternatively, elemental analysis can be used, as techniques like SEM-EDS will record high signatures of sulfur and nitrogen for protein-based fibers.^17^ For archeological fibers, where there is potential damage to morphological features, and a desire to avoid the sampling needed for SEM-EDS, a rapid noninvasive characterization can instead be performed using FTIR. In particular, animal hair proteins will have amide bands (ν(C = O) amide I at 1610–1650 cm^–1^ and ρ(N–H) amide II at 1515–1540 cm^–1^), while plant fibers will show peaks for cellulose (such as the C–O–C glycosidic bands at 1020 and 1105 cm^–1^ and a band at 3300 cm^–1^ from −OH stretching). IR spectroscopy allows for fibers to be sorted as either plant-based or animal hair, and from there more invasive characterization can be done only on the fibers of interest.

While aging can make animal hairs difficult to match to particular species by simple morphological traits or examination under a microscope, there is still a desire to better understand the source origins of these materials, for historical context (e.g., to understand ancient ecology and trade networks), to confirm or corroborate purported attribution, to prevent adulteration for animal fibers of high commercial value and to potentially flag the presence of material from endangered species (which has legal ramifications for the possession of such artifacts). Because animal fibers contain keratin as a primary component, an alternative identification scheme is to use mass spectrometry. In particular, the exact amino acid sequences present can be inferred by digesting a larger protein using an enzyme like trypsin and recording the different mass-to-charge ratios of the resulting fragment peptides. Because peptide sequences can differ between species, the recovered sequence information can be compared to existing protein sequencing data (where predicted masses can be compared to the measured *m*/*z* values) or previously measured mass spectrometric data for the species of interest. Once species specific markers are established (and ideally incorporated into an accessible database or repository), these can be used by subsequent researchers for species identification with mass spectrometry. Up until now, much of the focus of proteomics work has been on large Eurasian mammals,^39^ with few marker peptides identified for other animals of interest to cultural heritage research. As the field grows, additional protein sequencing information and peptide markers are being recognized and made available, suggesting that the technique will soon be viable for both quantitative and qualitative characterization of proteins in cultural heritage objects. Already a proteomics approach to fiber species identification has been successfully used to distinguish between Bovidae species^40^ and between Bovidae and Camelidae.^41^

In order to perform proteomic analysis, the proteins found in animal hair first need to be extracted and isolated. In the case of hair fibers, this can be challenging, as the proteins in hair are highly insoluble due to the formation of cross-linking, or disulfide bridges, covalently bonding sulfur-containing cysteine side chains across keratins and keratin associated proteins. Cross-linking helps confer good stability against chemical degradation in burial environments (with primary damage occurring due to the action of specific keratinolytic microorganisms), aids in mechanical strength (preventing breakage) and improves thermal stability. The consequence, though, is that hair samples are difficult to analyze due to their resistance to enzymatic cleavage. Prior to analysis, proteins need to be denatured to break the cross-linked network and solubilize the protein material. This can be done using a chaotropic agent (e.g., urea, ammonium bicarbonate, guanidine or thiourea) often paired with an alkaline compound capable of reducing thiol groups and breaking the disulfide bonds. These can include 1,4-dithiothreitol (DTT) or tris(2-carboxyethyl)phosphine hydrochloride (TCEP-HCl)). The first is particularly useful for extracting keratin from the cortex, while the second has been shown to efficiently extract keratin from the exocuticle.^42^ After breaking disulfide bonds through reduction, an alkylation step is needed to prevent reoxidation and is usually followed by a cleaning step (e.g., through ultrafiltration or solid-phase extraction) to wash away any remaining detergents, reduction/alkylation reagents and salts before the peptides are measured by MS.

In this work, we chose to evaluate two distinct extraction protocols. The first, referred to as the direct digestion method, uses a brief immersion in isopropanol and ammonium bicarbonate, while the second, abbreviated as the urea protocol, uses urea to denature the protein and TCEP as the reducing agent. The urea protocol is combined with a bead-based SP3 method adapted for the purposes of protein extraction for hair analysis. SP3 beads are carboxylate-modified magnetic beads that bind to proteins in order to isolate them from the buffer solution. Magnetic beads in a 1:1 ratio of hydrophilic to hydrophobic beads were mixed with the sample material and trypsin added within the same Eppendorf tube for digestion. Reducing the number of steps in this way helps to ensure that sample material is not lost during the extraction procedure.

## Experimental Procedures

### Materials

A selection of nine Chancay textile fragments, ranging in size from 2.5 × 6 cm to 19.7 × 13 cm, were chosen for analysis. The unfinished edges on all of the samples indicate that these were cut sometime after their discovery, since Chancay textiles were always woven to shape in order to preserve the life force of the textile.^43^ Many ancient Andean textiles that were uncovered in a burial context were fragmented. This was in part due to the actions of those who discovered the graves, as larger textiles were cut into multiple pieces to increase sales for dealers or to trim away damaged parts of textiles to make the artifacts appear more attractive.^44^

The fragments depict a variety of geometric patterns, small vertically and horizontally repeated motifs and zoomorphic figures, which are common designs attributed to the Chancay people. Of particular relevance to this study, the samples contain fibers dyed green, blue, red, yellow and black. Technical photographs and examination under optical microscopy were performed on all nine fragments to establish the weave structure. For further identification of the dyes and fibers used, short lengths of fiber were cut using methanol-cleaned scissors and weighed on a piece of glassine weighing paper. In all cases the extracted samples weighed less than 2 mg.

### Sample Preparation (Extraction Protocols)

Three different dye extraction protocols and two different protein extraction protocols were employed to isolate the dye and protein material for LC-MS analysis.

#### Direct Digestion^45^

Short lengths of fibers measuring a few millimeters (0.1–1.5 mg) were placed in a 1.5 mL Eppendorf tube with 100 μL of isopropanol and left in a thermomixer at 700 rpm and a temperature of 50 °C for 30 min. The supernatant was then removed and discarded, followed by the addition of 100 μL 50 mM ammonium bicarbonate. After vortexing for 1 min, the supernatant was removed and discarded and 15 μL of 20 μg/mL trypsin in 50 mM ammonium bicarbonate (1/2:1/2) was added to each sample and incubated at 37 °C for 3 h. The solution was evaporated to dryness using a Speedvac and resuspended in 10 μL of water with 1 μL of TCEP and CAA, followed by heating at 90 °C at a shaking speed of 10,000 for 10 min. The samples were then centrifuged at 17,000 xg for 4 min. Empore C18 stage tips (3M) were made in-house and used for desalting and concentrating the retrieved peptides, which were subsequently eluted using a solution of 80% acetonitrile, 0.1% formic acid.^46^

#### Urea Protocol

Approximately 1 mg of sample material was solubilized in 100 μL of 8 M Urea, 50 mM Tris HCl, 50 mM TCEP and 1 mM Na_4_EDTA at pH 8 and placed in the thermomixer overnight. The next morning, 25 μL of the same solution of 8 M Urea, 50 mM Tris HCl, 50 mM TCEP and 1 mM Na_4_EDTA was added and the sample left in the thermomixer for an additional 2 h.^47^ The solution was then spun down in a centrifuge at 17,000 xg for 5 min and a supernatant of 100 μL was transferred to a new Eppendorf tube to which 10 μL of 400 mM CAA was added for alkylation. The solution was left in the dark at room temperature for 45 min. To separate the dye material from the protein, μSPE was performed. In short, Pierce C18 pipet tips were activated by flushing 100 μL MeOH for three cycles of loading and unloading, followed by conditioning through flushing 100 μL of 8 M Urea, 50 mM Tris HCl, 50 mM TCEP and 1 mM Na_4_EDTA, again for three cycles.^47^ The sample material was then passed through the prepared tips for five cycles, after which the tips were washed with 0.1% formic acid for three cycles and, lastly, elution was done by flushing the tip with 40 μL MeOH:H_2_O:FA, 80:15:5 for five cycles. The eluted material was dried down and saved for the dye analysis. After removing the dye with μSPE, the SP3 protocol^48,49^ was used to aggregate the protein material onto magnetic beads, leaving behind any excess matrix material. For SP3, 20 μL of Sera-Mag A and 20 μL of Sera-Mag B were combined with 160 μL of water and incubated on a magnetic rack for 2 min. The beads were rinsed with 200 μL of water by pipet mixing and then allowed to settle against the tube wall (this preparatory step was repeated twice). Ten μL 100 mM TCEP/400 mM CAA were added to the sample for reduction/alkylation. After incubation at 95 °C for 10 min, 10 μL of the bead solution and 120 μL of 100% ethanol were added and mixed. Following an additional 10 min of incubation at room temperature, the solution with the sample material and SP3 beads was placed on the magnetic rack and allowed to settle, so that the supernatant could be removed and discarded. The sample tube was then removed from the magnetic rack and 200 μL of 80% ethanol used to rinse the beads, with the supernatant discarded after the beads settled. This was repeated twice. The aggregated protein was then digested using 10 μL trypsin (0.04 μg/μL) and 90 μL ammonium bicarbonate incubated at 37 °C overnight. The next morning, the sample was spun down in the centrifuge at 17,000 xg for 1 min, and the supernatant recovered and transferred to a fresh 1.5 mL Eppendorf tube. Empore C18 stage tips (3M) were made in-house and used for desalting and concentrating the retrieved peptides, which were subsequently eluted using a solution of 80% acetonitrile, 0.1% formic acid.

#### FA/MeOH Extraction

Fragments of the textile fibers with colors of interest were sampled and weighed (between 0.1 and 1.5 mg) and were immersed in 400 μL of formic acid/methanol (5:95, v/v) following Zhang and Laursen.^30^ The samples were incubated at 40 °C for 30 min, then spun down in the centrifuge at 1000 xg for 1 min. 320 μL of supernatant was removed and dried down in vacuum for 45 min at 45 °C.

#### Isopropanol and Ammonium Bicarbonate Extraction

The residue from the FA/MeOH solution was subjected to a second extraction protocol using isopropanol and ammonium bicarbonate. This involved taking the remaining fiber and FA/MeOH extraction solution and heating it at 50 °C for 30 min at a shaking rate of 700 rpm. The supernatant was then discarded and 100 μL of isopropanol added and placed back in the thermomixer for another 30 min of shaking at 700 rpm and 50 °C. The supernatant was removed and discarded and 100 μL of 50 mM ammonium bicarbonate added and incubated overnight at 37 °C, before the sample was evaporated to dryness in a vacuum desiccator.

### ATR-FTIR

ATR-FTIR was performed on all the textile samples to determine whether the fibers were of plant or animal origin. Preliminary infrared spectra were collected using a Thermo Nicolet 6700 Fourier Transform Infrared Spectrometer with an external Golden Gate single reflection micro attenuated total reflection accessory (ATR-FTIR) (incidence angle of 45°). A small piece of aluminum foil was used to cover the diamond crystal to protect it from contamination and the textile fragment was then pressed between the anvil and ATR top plate. The spectra were collected using a DTGS-KBr detector for a scan range of 400–4000 cm^–1^ at 4 cm^–1^ spectral resolution and averaged across 64 scans. Spectra were plotted as absorbance and matches were assigned by searching against the IRUG (Infrared and Raman Users Group) library.

### DART-MS

Direct Analysis in Real Time was performed using a DART 100 probe with a simplified voltage and pressure (SVP) controller (IonSense, Saugus, MA). The probe was positioned in front of an Orbitrap Elite mass spectrometer (Thermo Fisher Scientific, Waltham, MA) with a spacing of 7 mm between the ceramic cap of the DART and the transfer tube. Supplementary pumping (3.4 L/min) was provided by an IonSense Vapur flange fitted over the instrument inlet. Samples of fibers measuring a few millimeters were carefully extracted from the textiles and held with methanol-cleaned fine tip tweezers in the gap between the DART ionization source and the transfer tube. The DART helium temperature was set to 350 °C, and mass spectra were collected alternately in positive ion mode and negative ion mode, with the samples exposed to the heated gas plume for less than 1 min. Data were collected for short periods both before and after introducing the sample to the gas plume in order to collect background signal for comparison. Spectra were collected across 100–600 m/z at a resolving power of 120,000. Observed ions had the form of protonated [M + H]^+^ ions in positive mode or deprotonated [M − H]^−^ ions in negative mode.

### LC-MS

After dye and protein extraction, 10 uL of sample material was resuspended in 0.1% formic acid and injected onto ThermoScientific Acclaim PepMap 100 trap columns (100 μm i.d. x 2 cm, 5 μm particle size) and separated on a ThermoScientific Acclaim PepMap RSLC analytical column (75 μm i.d. x 25 cm, 2 μm particle size) at 300 nL/min with the following gradient: 0–2 min, 2% B; 2–38 min, 2–31% B; 38–40 min, 31–90% B; 40–43 min, 90% B; 43–44 min, 90–2% B; 44–60 min, 2% B. Buffer A was 0.1% Optima formic acid (FisherScientific) in Optima water, and Buffer B was 0.1% Optima Formic Acid in Optima acetonitrile. Data were collected on a ThermoScientific Orbitrap Elite as data dependent analysis (DDA) with the following parameters: MS1 was collected from 375 to 2000 *m*/*z* at 60000 resolving power with 1E6 AGC and 100 ms max inject time. A top 10 method was used with the following parameters: 15000 resolving power, 30% NCE HCD, default charge state 2, 0.1 ms activation time, 5 *m*/*z* isolation width, fixed first mass at 100 *m*/*z*, 5E5 AGC, and 250 ms max inject time. In between each sample run a wash was included to minimize carryover.

### Data Analysis

Mass spectra were processed using Thermo XCALIBUR controlling software (USA).

### Species Identification

RAW files were searched with MetaMorpheus 1.0.5^50^ against databases for Ovis aries, Lama, Vicugna, Laurasiatheria, Bovidae and Artiodactyla downloaded from Uniprot (2024). The calibration, G-PTM-D and search task parameters were all set to two maximum missed cleavages, a minimum peptide length of seven and a variable initiator methionine behavior. For modifications, carbamidomethyl on C or U were set as fixed and oxidation on M was set as variable, with a maximum of two modifications allowable per peptide and a value of two input for the max modification isoforms. The total G-PTM-D modifications count was set to 60. For both the G-PTM-D and search task the precursor ions were assigned a 5 ppm mass tolerance and the product ions a 20 ppm mass tolerance. For the search task, a 1% false discovery rate (FDR) was used, and the identified peptides were queried against the NCBI protein database and Uniprot database using the basic local alignment search tool (BLAST) to determine the taxa corresponding to the recovered peptide sequences. The mass spectrometry proteomics data have been deposited to the MassIVE repository with the data set identifier MSV000095885.

### Dye Compound Identification

Skyline was used to search the spectra for candidate protonated or deprotonated ions based on a list of potential dye compounds. Dye compounds found in the literature were input to the software as a table of compound name, molecular formula and precursor ion charge (Table S1). Skyline then tagged compounds that met a prescribed threshold signal intensity. Compounds were considered to be present if the signal intensities were higher than 10^3^ and if the C_13_ and C_12_ isotopic peaks showed the expected behavior. A summary of the identified marker compounds is provided in the supporting materials (Table S2).

## Results and Discussion

### Establishing Fiber Type

For all of the textile fragments under consideration, ATR-FTIR spectra were collected on multiple regions where there were visible differences in the colors used. Searching the spectra against libraries produced matches for cellulose for six of the samples and protein-based fibers for five of the samples. The results are summarized in Table S3. For colored regions where the ATR-FTIR spectra matched protein-based fibers, small lengths of fibers were sampled for further MS analysis of the dyes and proteins present.

### Mass Spectrometry Dye Extraction

#### Blue Dyes

**Figure 1:**
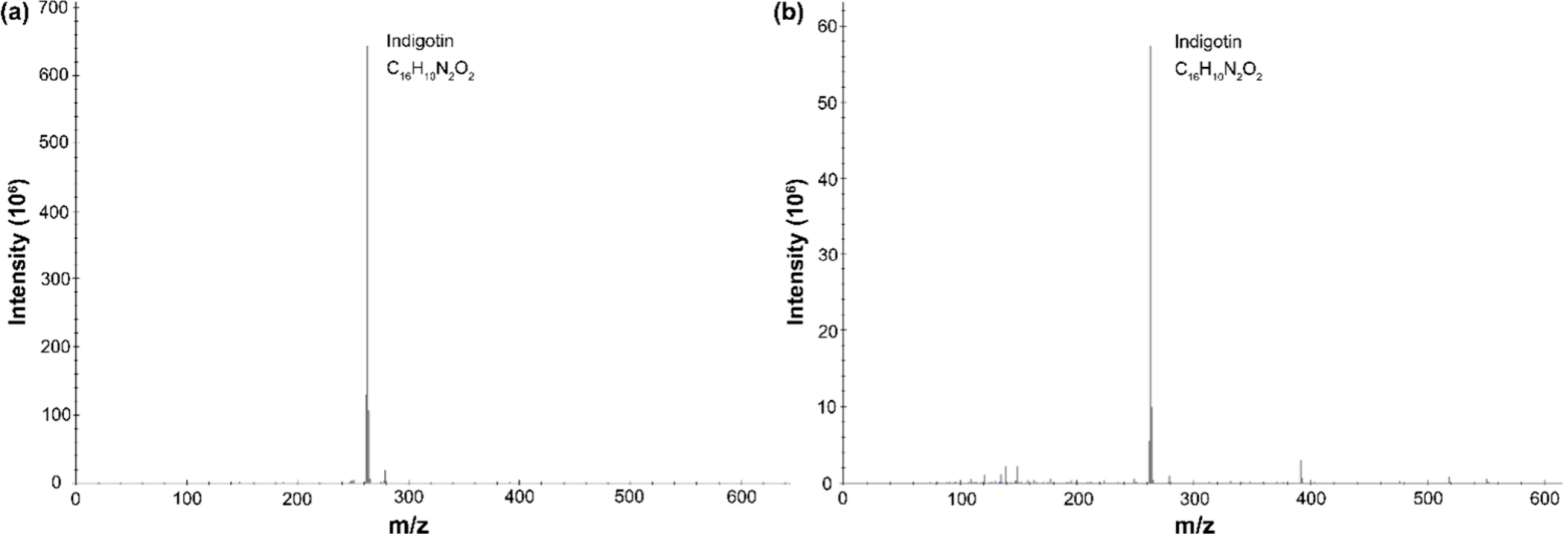
Positive ion mode DART mass spectra for blue colored fibers from (a) P.6862.1.1. and (b) P.6862.1.8.

Both of the blue fibers sampled produced mass spectra consistent with indigo when measured by DART-MS, as shown in Figure 1. The primary marker for indigo is indigotin, which appears at *m*/*z* 263.083 Da and was found in both samples (Figure 1), with additional marker molecules like 2-indoline, caffeic acid, indoxyl acetate and isatin. Some small differences were apparent between the two polarities measured, with indican appearing only in positive ion mode for sample P.6862.1.8. Additionally, there were no peaks corresponding to 6,6′-dibromoindigotin, making it possible to rule out blue dyes created from a brominated indigoid, like shellfish (e.g., *Concholepus concholepus* or *Thais chocolata*), and suggesting instead the use of an indigo producing plant. For pre-Columbian textiles, the likely species of indigo plants would be from Indigofera (e.g., *I. suffructicosa* and *I. truxillensis*), Eupatorium (e.g., *E. glechonophyllum* and *E. salvia*) or Cybistax (e.g., *C. antisyphilitica*).

The use of complementary techniques can be particularly relevant for compounds like indigoids, which exhibit different ionization behaviors. Specifically, it has been reported that indigotin is difficult to ionize by protonation with electrospray,^51^ potentially due to internal hydrogen bonding. For this reason, DART-MS proved particularly useful for the rapid identification of indigo in the blue, green and black fibers.

#### Red Dyes

LC-MS results for the red dyes from sample P.6862.1.5 and P.6862.1.9 are presented below. The chromatogram profiles show that the extracted dye compounds separated into two groups, one at a retention time around 9.81 min and the other around 18.51 min. The first major peak, centered at 9.81 min, corresponded to a mass spectra with a dominant peak for the [M-H]^−^ ion at 491.0831 *m*/*z*, consistent with carminic acid. Appearing slightly before this peak was a small peak at 9.66 min, whose mass spectra matched dcII. The second elution event was composed of three large overlapping peaks. These could be distinguished by their mass spectra, which revealed precursor ions at 313.0354 *m*/*z*, 329.0303 *m*/*z* and 269.0455 *m*/*z*. Matches were found for laccaid acid D, kermesic acid and lucidin, respectively. A relatively minor peak was found offset at a retention time of 18.27 min, which could be assigned to rubiadin. Other low intensity peaks were matched to xanthopurpurin or alizarin, purpurin and pseudo purpurin. The dominant peak for carminic acid points to the use of cochineal dye, whose marker compounds also include dcII, laccaic acid D and kermesic acid. In Peru, the scale insect likely used for dyeing was *Dactylopius confucius* or *Dactylopius coccus*.

The presence of purpurin, xanthopurpurin or alizarin, pseudo purpurin and lucidin could suggest that plant-based anthraquinone dye was used as well, likely from a genus within *Rubiaceae*, like Relbunium or Galium. In particular, the genus *Relbunium*, which contains several plants historically used for dyeing, is indigenous to Central and South America (with 25 species across the Americas and 7 species within Peru). The Relbunium plants potentially used in Peruvian dyeing include *R. ciliatum*, *R. microphyllum*, *R. nitidum*, *R. gibemium*, *R. tetragonum*, *R. hirsutum* and *R. richardianum*.^52^

Previous authors have identified marker compounds from both animal- and plant-based anthraquinone dyes in individual red fibers, suggesting that mixtures were formed during the dyeing process. In Peru, the type of red dye used is usually taken as a chronological marker, with *Relbunium* dyes dominating during the Late Formative and Early Middle periods when the Paracas and Nasca cultures existed (1100 B.C.-600 A.D.). By the Late Middle Period (900–1470 A.D.), these plant-based dyes had been largely displaced by cochineal red, which was preferred by the Chimu and Moche cultures.^2,53^ The cochineal insect was first imported from Mexico sometime in the Early Intermediate Period (200–600 A.D.), possibly becoming established after this point due to a change in climate that permitted the cultivation of the *Opuntia exaltada* cactus that the insect feeds on.^52^ Beginning in the years 600–900 CE, both plant and animal based anthraquinone dyes were used,^53^ making it likely that the red dyed textiles studied here were created relatively early in the Late Intermediate Period.

Little difference was seen between the LC profiles for the three different dye extraction methods used on the red fibers, with the major compounds of carminic acid and laccaic acid D appearing in all cases. For the urea extraction, the overall peak intensities were lower, and some minor components, like alizarin (for P.6862.1.9) and lucidin (for P.6862.1.5), failed to be detected.

Some of the anthraquinone aglycones, like laccaic acid D, alizarin and purpurin, were identified by DART-MS, as well (negative mode DART-MS for P.6862.1.9 and positive mode DART-MS for P.6862.1.5), though carminic acid was conspicuously absent. The difficulty in detecting glycosides like carminic acid is presumably due to its low volatility. Armitage et al.^16^ have suggested that this is due to the strength of the hydrogen bonding of the hydroxyl groups within the glucose moiety, which resists desorption by the heated gas plume.

**Figure 2:**
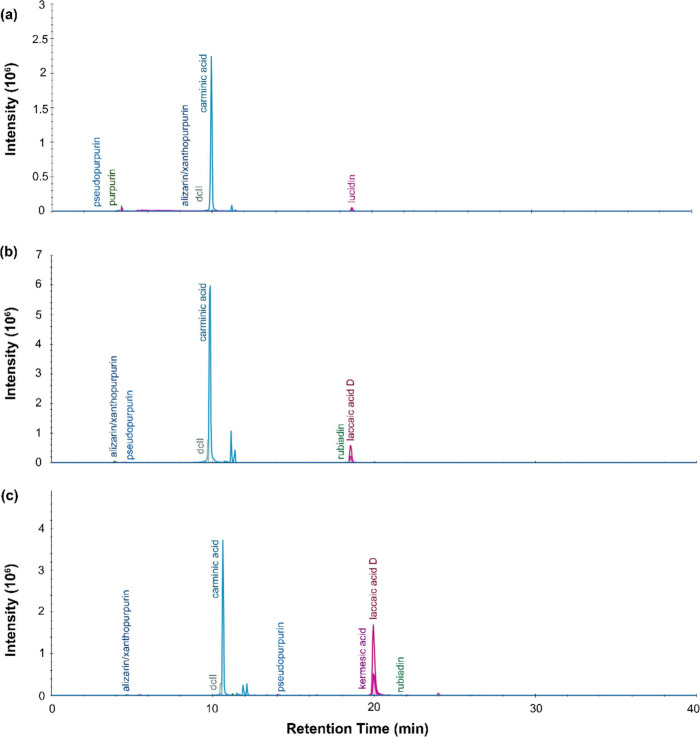
LC-MS chromatogram profiles for red dye material from P.6862.1.5 extracted with (a) urea protocol, (b) isopropanol and ammonium bicarbonate protocol, and (c) FA/MeOH protocol.

**Figure 3:**
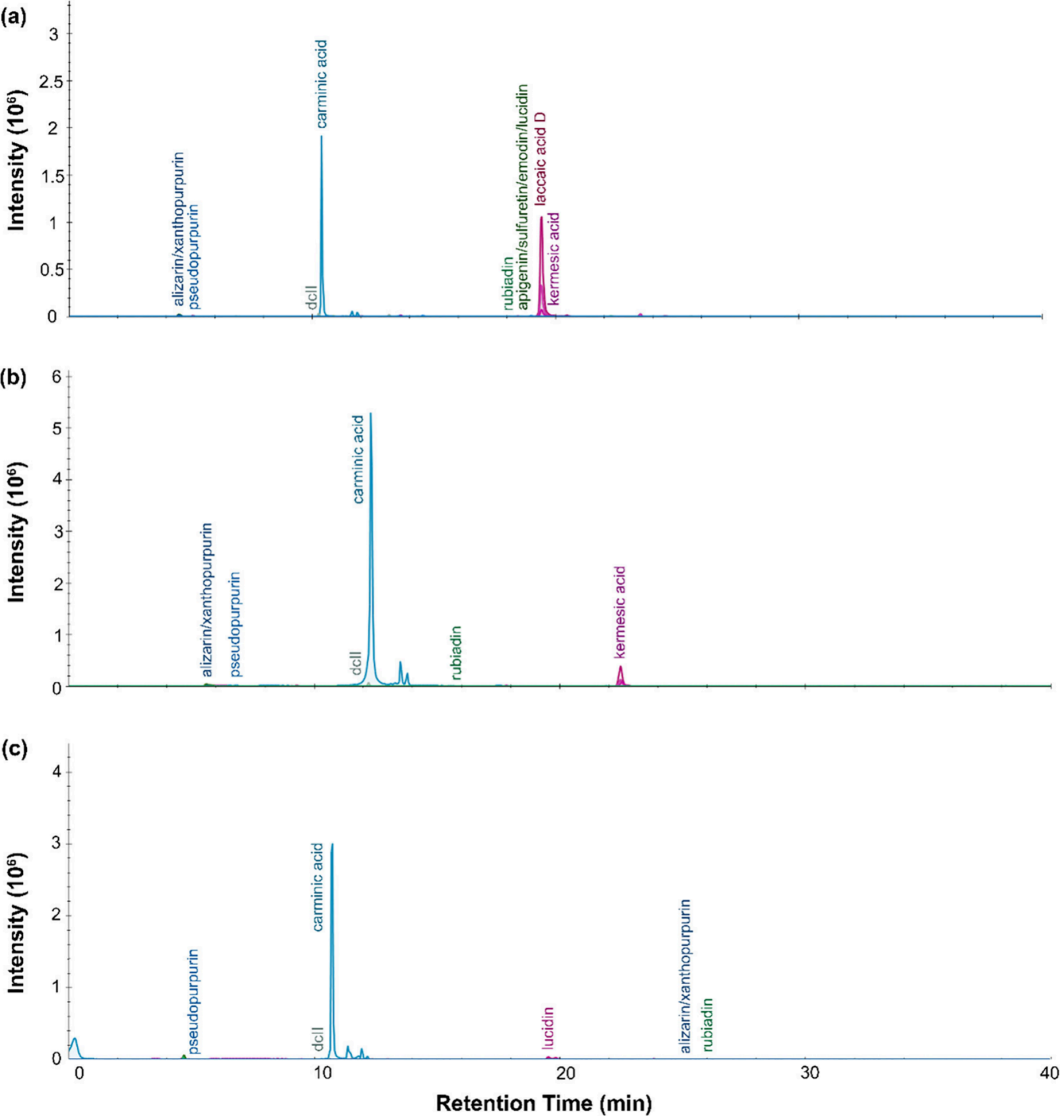
LC-MS chromatogram profiles for red dye material from P.6862.1.9 extracted with (a) urea protocol, (b) isopropanol, and ammonium bicarbonate protocol and (c) FA/MeOH protocol.

#### Yellow

There were a limited number of natural sources for red and blue dyes used by ancient Andean dyers, but the number of plants that produce yellow compounds is extensive. This is because yellow color comes most frequently from flavonoid molecules, which are represented widely across plant families. Beyond flavonoids, there are additional chromophores in plants that can create yellow, including carotenoids, alkaloids and curcuminoids. The extreme biodiversity of yellow dye plants, together with their high photosensitivity, means that proper identification of yellow dyes in ancient textiles is extremely challenging. While identifying yellow dye plants is difficult, it also implies that when successfully identified, the yellow dye plants described can offer valuable information about the resources available. The following have been suggested as potential plants used for yellow dyes in pre-Columbian Peruvian textiles: *Bidens andicola* (along with other Asteraceae like *Coreopsis*, *Cosmos* and *Dahlia*), *Baccharis* species (*B. floribunda* and *B. genistelloides*), *Alnus jorullensis*, *Kageneckia lanceolata* and *Hypercium larcifolium*. Given its ability to handle complex matrices of chromophores, LC-MS is the premier technique for analyzing flavonoid compounds.^20^

Four of the textile fragments considered in this work contained yellow dyed wool. DART-MS was relatively unsuccessful for these samples, with the primary ions corresponding to hydroxybenzoic acids (4-hydroxybenzoic acid at 137.0244 *m*/*z*, 4-hydroxy-3-methoxybenzoic acid at 167.0350 *m*/*z* and 3,4-dihydroxybenzoic acid at 153.0193 *m*/*z*). Three of the samples (P.6862.1.4, P.6862.1.5 and P.6862.1.6) also had peaks at 359.0722 *m*/*z*, which may be due to sideritiflavone/jaceidin/centaureidin, a minor component found in *Baccharis floribunda*, known locally as chilca.

**Figure 4:**
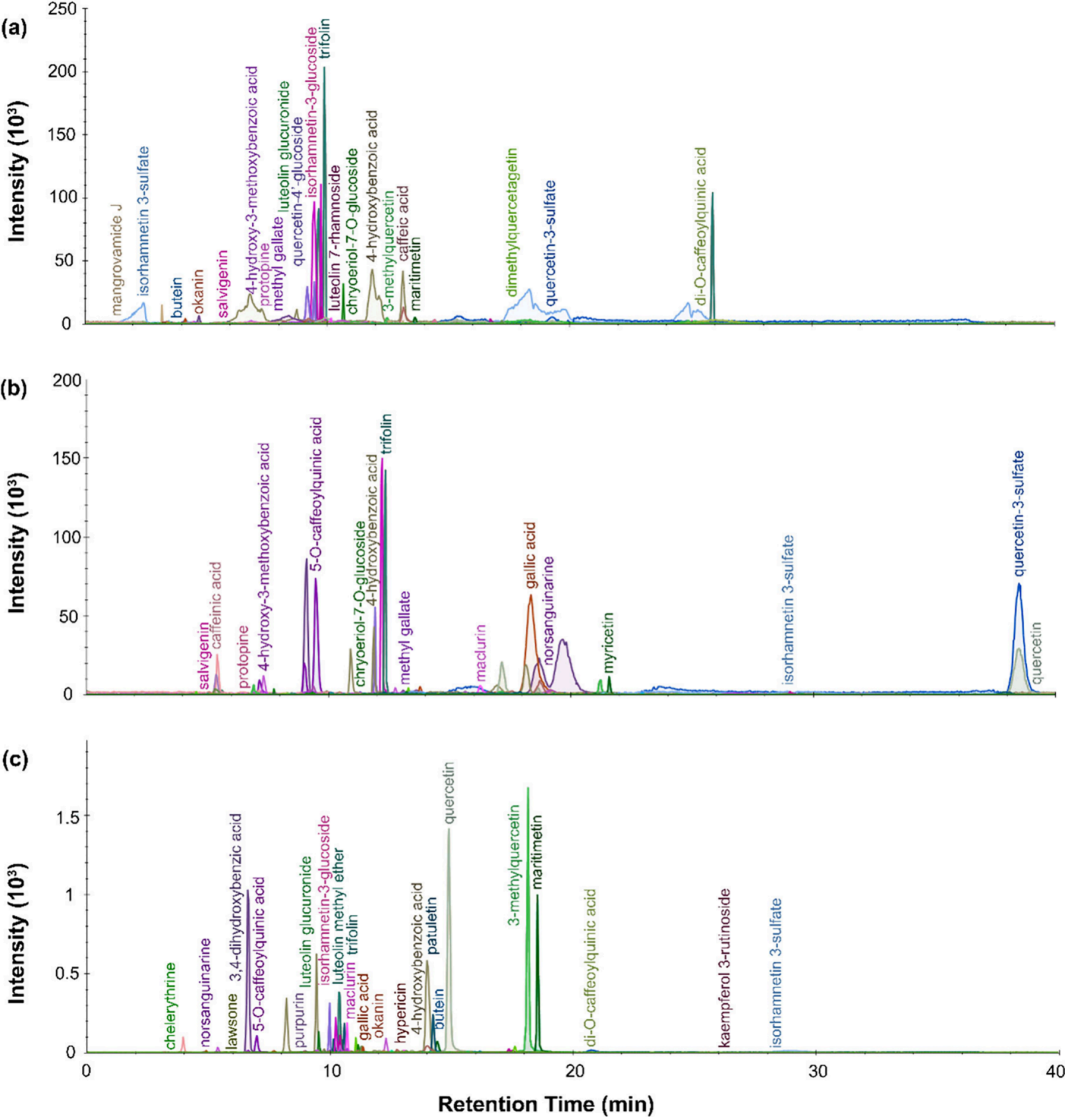
LC-MS chromatogram profiles for yellow dye material from P.6862.1.3 extracted with (a) urea protocol, (b) isopropanol and ammonium bicarbonate protocol, and (c) FA/MeOH protocol.

**Figure 5:**
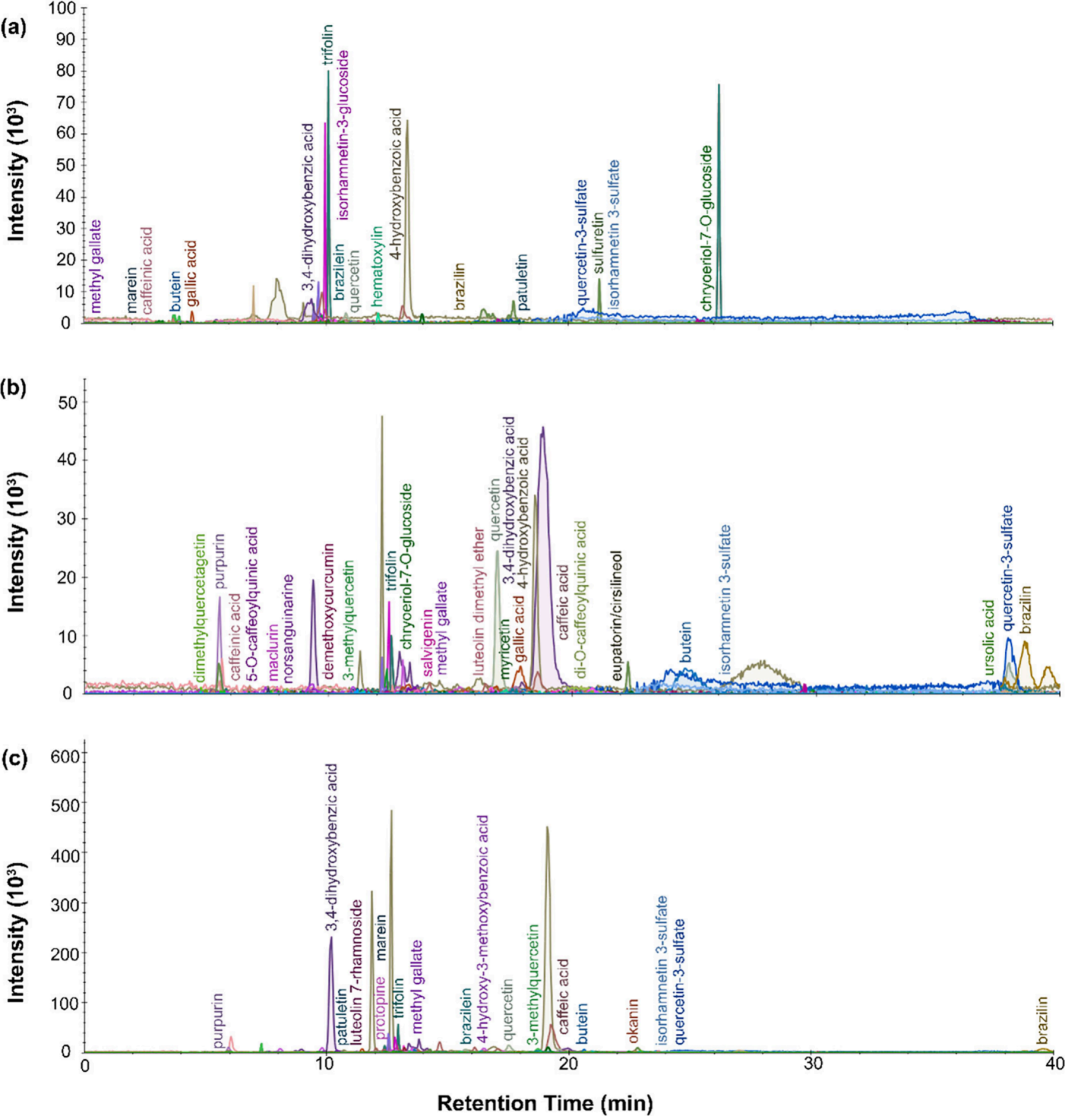
LC-MS chromatogram profiles for yellow dye material from P.6862.1.5 extracted with (a) urea protocol, (b) isopropanol and ammonium bicarbonate protocol, and (c) FA/MeOH protocol.

**Figure 6:**
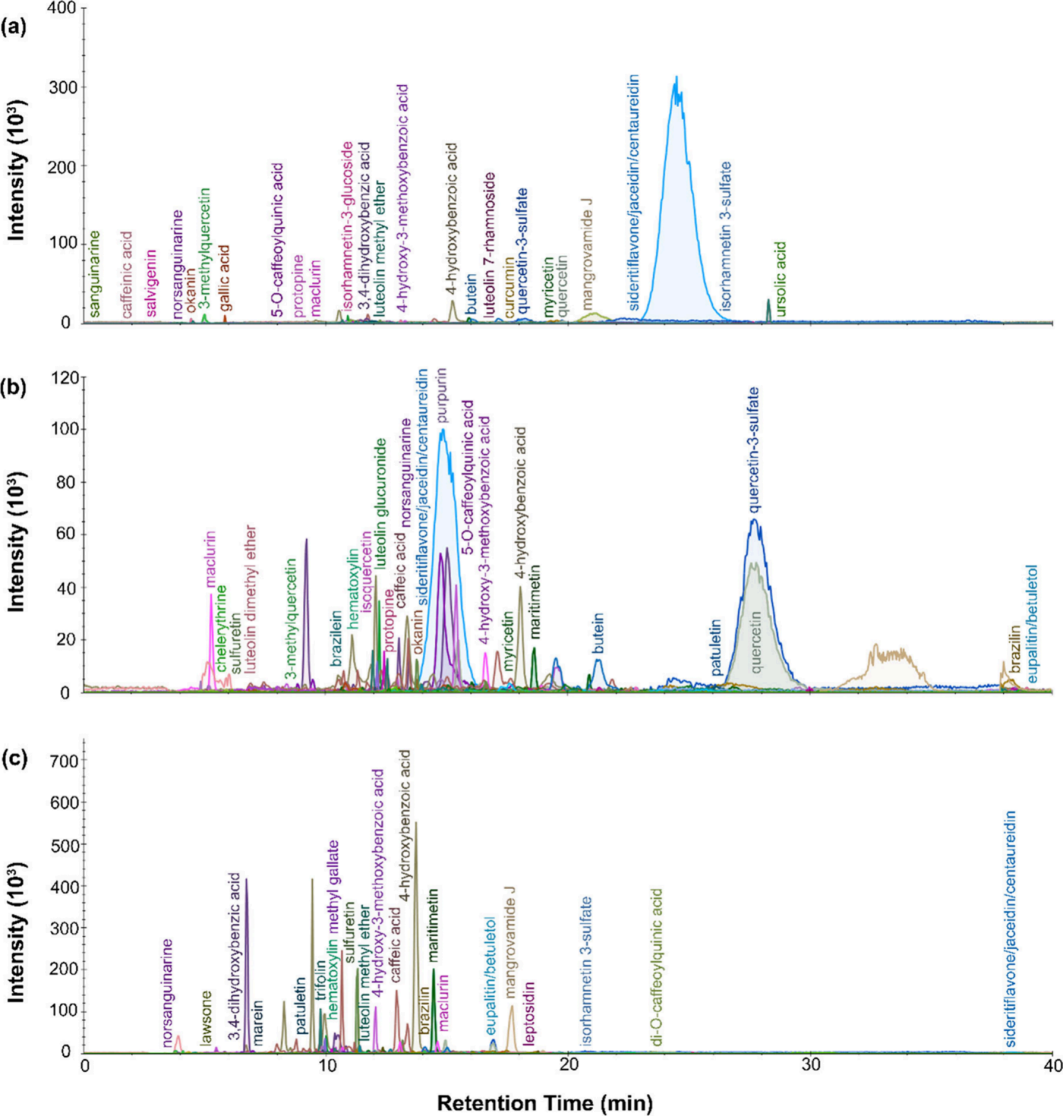
LC-MS chromatogram profiles for yellow dye material from P.6862.1.6 extracted with (a) urea protocol, (b) isopropanol and ammonium bicarbonate protocol, and (c) FA/MeOH protocol.

**Figure 7:**
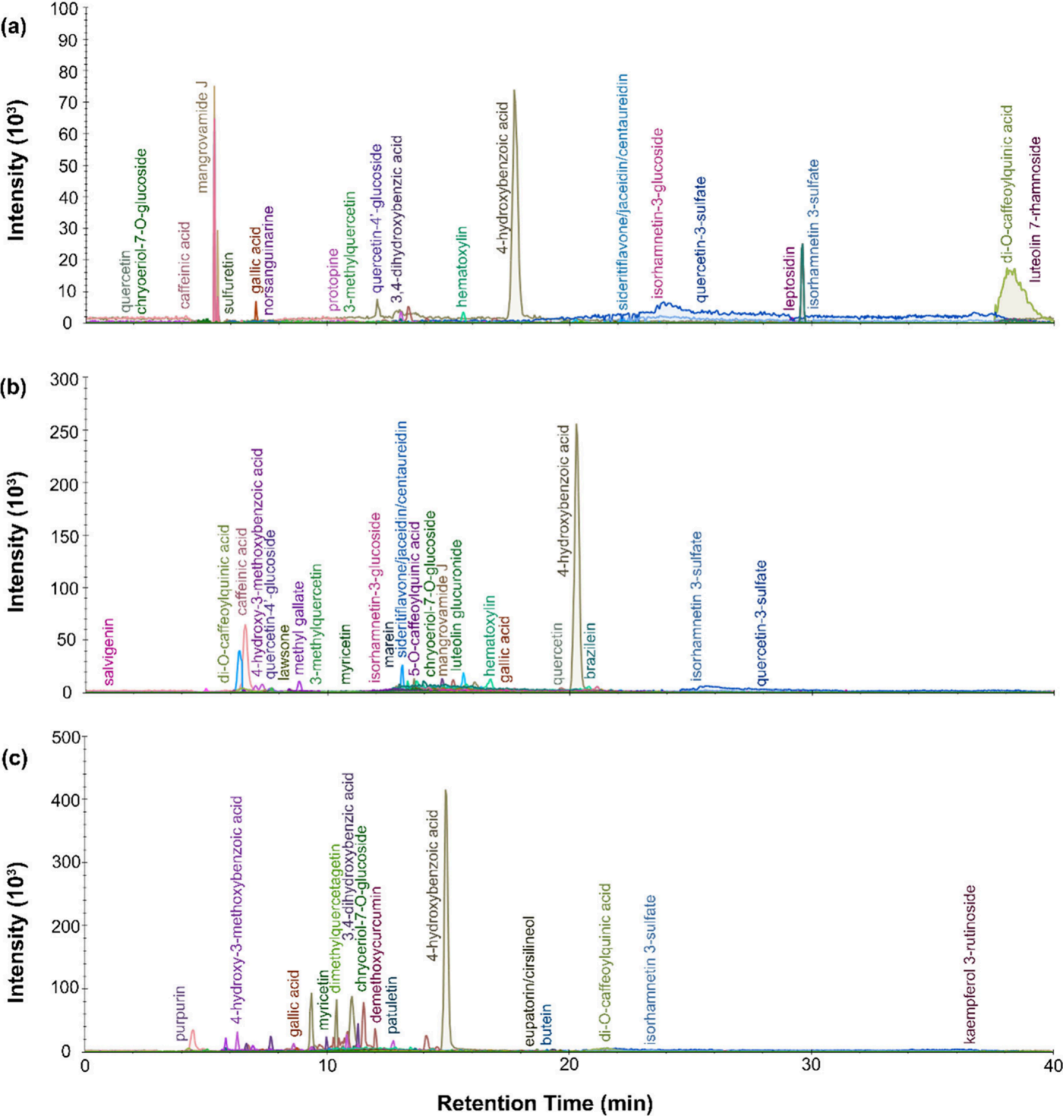
LC-MS chromatogram profiles for yellow dye material from P.6862.1.9 extracted with (a) urea protocol, (b) isopropanol and ammonium bicarbonate protocol, and (c) FA/MeOH protocol.

From the LC-MS results, presented in Figures 4, 5, 6, and 7, the chromatograms for the dye extracts resulted in a dense series of peaks. In the sample from P.6862.1.3, two very intense peaks dominate the LC profile. The first appears at around 14.9 min and has a mass spectrum that matches well with quercetin. The second comes at around 18.2 min, and its mass spectrum corresponds with a compound with a molecular formula of C_16_H_12_O_7_ ([M-H]^−^ at *m*/*z* 315.051), possibly due to tetrahydroxymethoxy-flavone. Closely following this peak is another at 18.5 min, assigned to maritimetin. The other high abundance compounds include hydroxybenzoic acids, 5-O-caffeoylquinic acid, quercetin-4-glucoside, isorhamnetin-3-glucoside, trifolin, maclurin, dimethylquercetagetin and patuletin. For P.68621.4, there are fewer high intensity peaks. Besides the signal for hydroxybenzoic acids, lower abundance compounds include okanin, butein, caffeic acid, quercetin and purpurin. A similar profile appears for the extract from P.6862.1.5, which primarily has hydroxybenzoic acid, along with smaller amounts of brazilin, okanin, purpurin, caffeinic acid, isorhamnetin-3-glucoside, trifolin, quercetin, butein, sulfuretin, marimetin, tetrahydroxymethoxy-flavone and gallic acid. For P.6862.1.6, the main compounds are hydroxybenzoic acids, mangrovamide J, sulfuretin, maritimetin, quercetin-3-sulfate, caffeinic acid, caffeic acid, trifolin, quercetin-4-glucoside and purpurin. Mangrovamide J was detected and can be used as a marker compound to differentiate *Cosmos sulphureus* from other chalcone-type plant dyes (e.g., *Bidens andicola*).

The dye material retrieved from P.6862.1.9 (Figure 7) produced less clear results overall, with significant broadening of peaks and noise in the chromatogram. From the LC-MS, the sample had hydroxybenzoic acids, caffeinic acid, caffeic acid, butein, gallic acid, di-O-caffeoylquinic acid and quercetin-4-glucoside.

#### Green

Of particular interest to art historians when considering ancient dyeing technology is determining the process used by dyers to create secondary colors. Primary colors include blue, red and yellow. Secondary colors like orange, green and purple are traditionally achieved by mixing or overdyeing. Secondary colors could potentially be achieved by overdyeing one primary color on another primary color, or on a naturally colored fiber, by the pairing of a primary color with a mordant or by mixing multiple dyes in a single dye vat. Especially in cases where only a single dye is detected, it is often difficult to determine whether a second dye was originally present and decomposed over time, or if the dyers achieved the observed color by adjusting dyeing conditions like dye bath temperature and choosing specific mordants.

There are almost no plant or animal products that yield a stable green color. As a result, green textiles often required the mixing of yellow and blue dyes or the application of blue dye onto a naturally yellow colored fiber.

**Figure 8:**
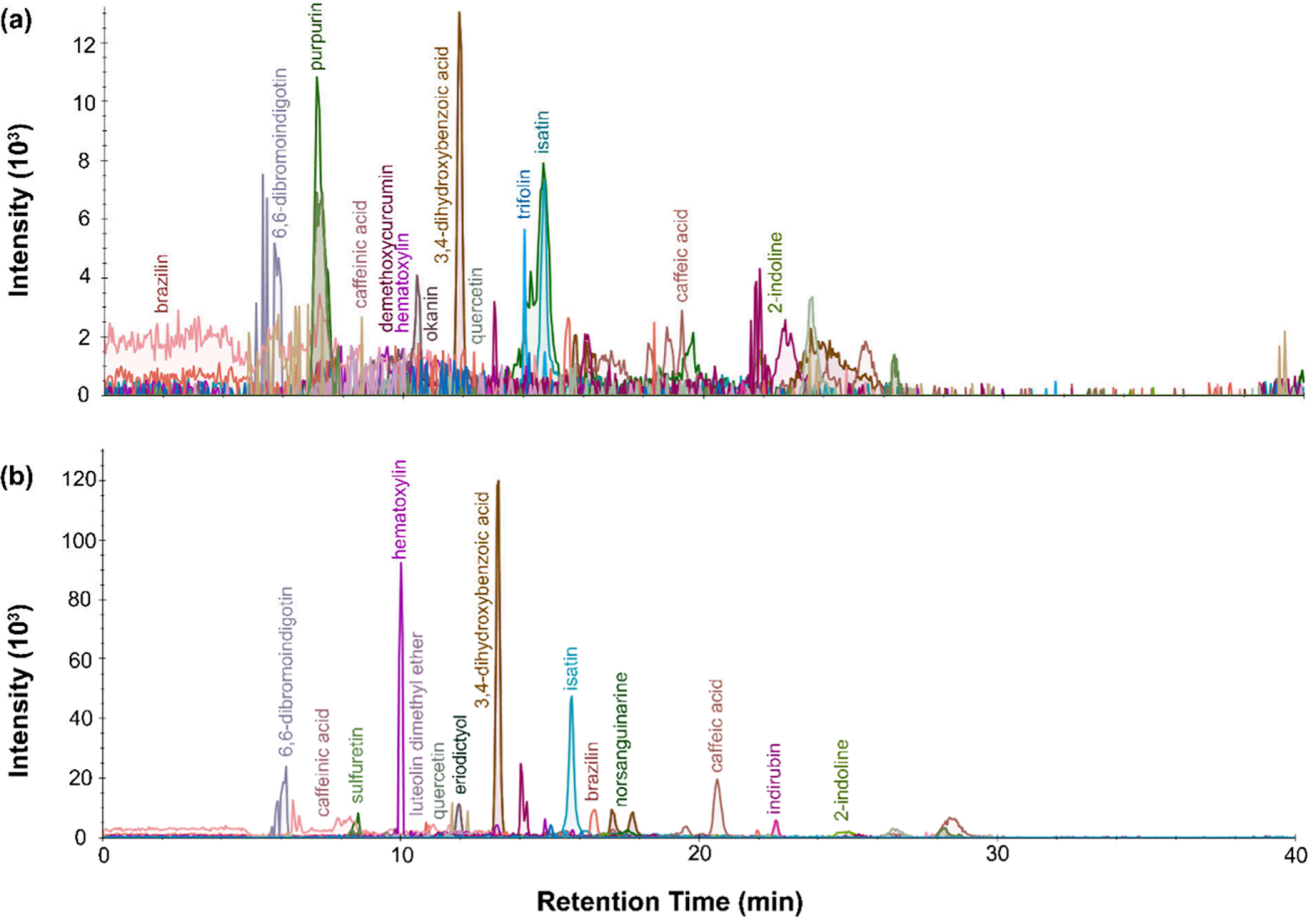
LC-MS chromatogram profiles for green dye material from P.6862.1.4 extracted with (a) isopropanol and ammonium bicarbonate protocol and (b) FA/MeOH protocol.

**Figure 9:**
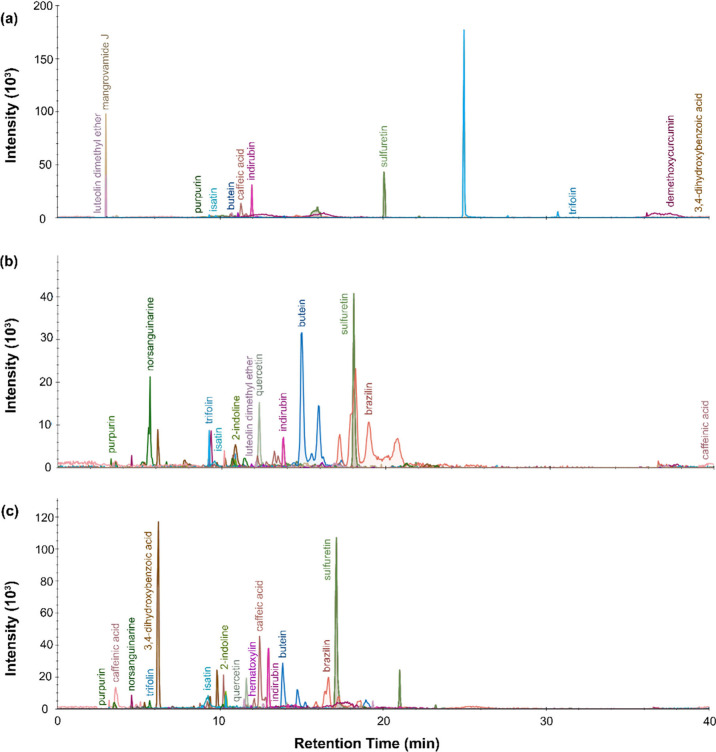
LC-MS chromatogram profiles for green dye material from P.6862.1.5 extracted with (a) urea protocol, (b) isopropanol and ammonium bicarbonate protocol, and (c) FA/MeOH protocol.

Only two of the textile fragments studied had areas of green dye. The results of the LC-MS measurements for these two samples are reported in Figures 8 and 9. Indigotin appeared in the DART mass spectra for both green samples (P.6862.1.4 and P.6862.1.5) in both polarities. However, some difference was seen in the extraction protocols used for LC-MS, with indigotin appearing only for the urea and MeOH extractions for P.6862.1.5, though isatin did appear in the urea extraction for P.6862.1.4. For the sample taken from P.6862.1.4, additional detected compounds included 3,4-dihydroxybenzoic acid, isatin, indirubin, caffeic acid, brazilin and okanin. At a retention time of around 10 min, a sharp peak appeared that was identified as hematoxylin. The presence of brazilin and hematoxylin point to the use of a redwood dye, while the chalcone okanin suggests the use of an Asteraceae plant, like *Bidens andicola*. The chromatogram profile for the fragment of green yarn taken from P.6862.1.5 is similarly complex, with a series of lower intensity peaks appearing within the first 25 min of elution. The relatively high number of peaks can be attributed to both the presence of a plant-based yellow dye, since these contain mixtures of chromophores, and the potential build up of degradation products. The formic acid/methanol dye extraction was chosen as representative of the dye composition, and the results from this dye extraction were used for identification purposes. Sulfuretin, 3,4-dihydroxybenzoic acid, caffeic acid, indirubin, butein and brazilin are the most pronounced peaks. The chalcone butein and the aurone sulfuretin are two of the primary colorants in *Cosmos sulphureus*, making it likely that this flower, or another related Asteraceae plant, was used. The presence of the homoisoflavonoid brazilin would indicate the additional use of a redwood dye, like *Caesalpinia* (Brazilwood). Though typically associated with a red color, the dye from *Caesalpinia* can also produce a yellow color. This flavonoid dye could have been sourced from either *Caesalpinia paipai* or *Caesalpinia spinasaare*, both of which grow in Peru. Multiple peaks were observed corresponding to hydroxybenzoic acid. These are the oxidation products of unsubstituted flavonol dyes (i.e., 3-hydroxyflavone dyes) and can be created specifically through the photo-oxidation of the C2–C3 bond in the carbon ring. Prior research has concluded that these small molecules are markers of aging, due to the fact that they are not wash-fast and thus cannot be a byproduct of the dyeing process. The 3,4-dihydroxybenzoic acid identified is thus likely a photo-oxidation product of quercetin, since quercetin is another marker compound associated with Asteraceae plants. Overall, the results for both green dye extracts suggest that Chancay dyers made use of flavonoid yellows, presumably from Asteraceae plants, in combination with redwood dyes, to create a base yellow color that could be overdyed with indigo to create green.

#### Black

**Figure 10:**
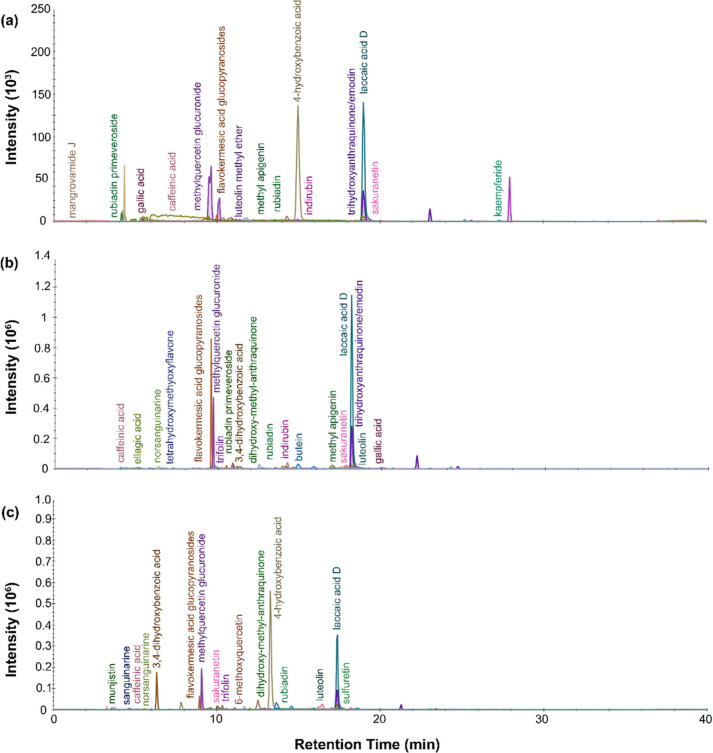
LC-MS chromatogram profiles for black dye material from P.6862.1.5 extracted with (a) urea protocol, (b) isopropanol and ammonium bicarbonate protocol, and (c) FA/MeOH protocol.

**Figure 11:**
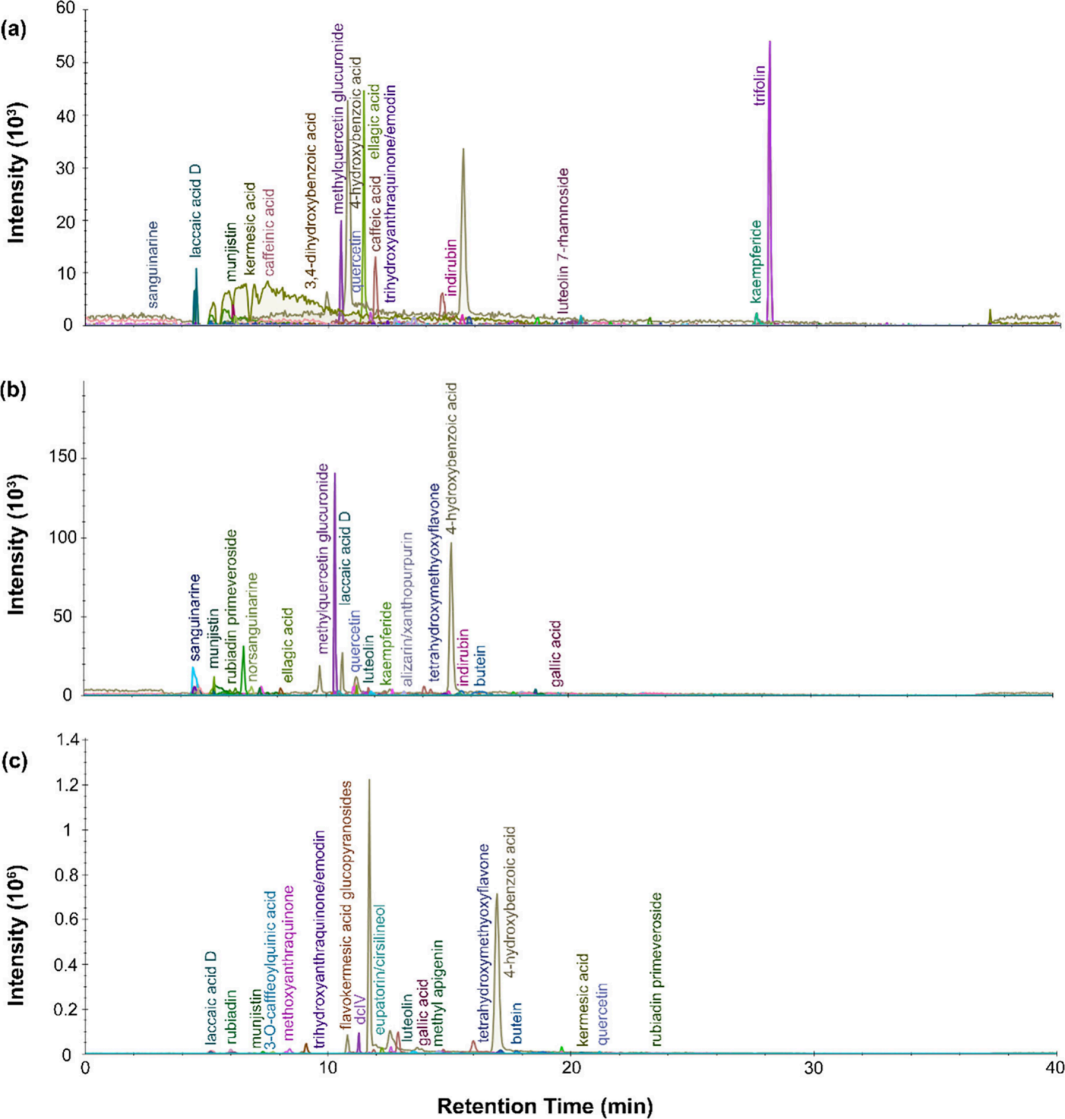
LC-MS chromatogram profiles for black dye material from P.6862.1.9 extracted with (a) urea protocol, (b) isopropanol and ammonium bicarbonate protocol, and (c) FA/MeOH protocol.

From the LC-MS chromatograms for the black dye extracts, multiple isolated peaks can be identified. For P.6862.1.5 (Figure 10), an intense peak appeared at around 17 min, which, based on the corresponding mass spectra, can be attributed to laccaic acid D. This, along with the detection of carminic acid, plus dcII and dcofk, two flavokermesic acid glucopyranosides, and dcVII (a β C-glucofuranoside of kermesic acid), indicates the use of cochineal dye. Additional compounds include hydroxybenzoic acids, emodin and caffeinic acid. Caffeinic acid can be found in walnut dyes, which generally were used for brown colors, and emodin and hydroxybenzoic acids both can be explained by a flavonol-containing dye. The dye extract retrieved from P.6862.1.9 (Figure 11) had fewer well-resolved peaks. The primary signal came from hydroxybenzoic acids, which could indicate a flavonoid dye, but could also come from degradation of the wool itself.^54,55^ As in the previous sample, some carminic acid and dcVII was identified, this time along with butein, caffeinic acid, caffeic acid, luteolin dimethyl ether, luteolin methyl ether, dihydroxy-methyl-anthraquinone, methoxyanthraquinone and indirubin. Of note, ellagic acid was also identified for this sample, suggesting the presence of tannins. The ellagic acid identified could point to a brown dye from a plant like *Schinopsis* or *Juglans* or a redwood like *Caesalpinia*. That said, ellagic acid can also be found in low abundance in other dye plants (including those that produce flavonols and anthraquinone dyes), like *Baccharis genistelloides*. The results from both black-colored fibers registered the use of a wide mix of colored compounds, including indigo-based blue, flavonoid yellows, anthraquinone reds and tannin-based browns. These results imply that rather than choose a naturally pigmented wool or produce black with a single dye like *Kageneckia* or logwood, the dyers opted to mix a wide variety of dyes to achieve a dark color. Given the diversity of chemical compounds, this could have involved mixing together the residual material from primary color dyes and repurposing it for creating black yarn.

### Species Identification for Protein-Based Fibers

The protein extracted from the animal hair-based fibers were measured using mass spectrometry for purposes of taxonomic identification. A proteomics approach using mass spectrometry relies on peptide sequence variation that can be linked to particular species. Provided that the protein containing the peptide of interest is extracted from the fiber and digested into appropriately sized fragments, the differences in amino acid residues will produce ions of different masses. Compared to techniques like microscopy, mass spectrometry facilitates the objective analysis of both chemically-treated and aged fibers and has been successfully applied to the taxonomic identification of historical textiles.^56−58^

The collected mass spectra were searched against multiple fasta files corresponding to different candidate taxonomic groups, and the complete peptide spectral matches are listed in the supplementary files (Tables S4 and S5). The recovered proteins were primarily type I and type II keratin, although additional material, including keratin associated proteins, ribosomal proteins and histones, was detected in some cases. Species identity was determined both using the BLAST alignment tool and based on the presence of marker peptides from the literature.

Some immediate conclusions can be drawn based on the keratin peptide sequences. All of the fiber samples investigated lacked the appropriate marker peptides for the genus *Ovis*, making it possible to rule out the use of sheep’s wool. This is in keeping with the fact that the Spanish introduced sheep to Peru in the 16th century,^59^ well after the disappearance of the Chancay. In particular, a type I K33b keratin protein has a sequence toward its C terminal end, which contains YSCQLSQVQSLIVNVESQLAEIR and has been cited in the past as a good marker for identifying both wild and domestic sheep.^60−62^ The animal hairs studied here had YGSQLSQVQGLITNVEHQLAEIR, instead, matching the *Lama* genus. Another sequence corresponding to a type I cuticular Ha3–1 keratin protein was measured as DVEEWFAR. This can also be used to differentiate between *Ovis* and *Lama*, since the sequence in *Ovis* has a threonine in place of an alanine. Some of the type II keratin markers measured also point to camelid species more broadly, including the presence of LAELEGALQK, LASELNHVQEVLEGYR and DLNLDCIVAEIKEQYDDIAR, the last of which was designated by Azemard et al.^62^ as a marker peptide sequence for camelid hair. Likewise, certain keratin associated proteins (KAPs) have sequences specific for Camelidae, as was found for KAP 3–3, which had a sequence of TGPATTICSFDK.

There are four camelid species of relevance to textile production in the ancient Andes. Two of these are wild species (vicuña and guanaco). Both were domesticated early on, leading to two domesticates (llama and alpaca). While there is significant interspecies similarity, the quality of the hair fibers produced by each of the South American camelids differs, making species identification useful for both fraud prevention and conservation efforts in modern day textile trade, as well as for better comprehending the structure of ancient cultures. Llamas typically have coarser hair than alpacas, and vicuñas have particularly fine hair. In the context of the social hierarchy of the Chancay, finer quality fibers would have likely been reserved for use by the highest social classes.^37^

Even in cases where the surface morphology is well preserved, the previously mentioned identification strategies, like SEM, are not well suited to differentiating between llama, alpaca, vicuña and guanaco, since all four have relatively similar cuticular cell morphology, generally described as having smooth cuticular cell margins and a fragmented medulla.^63^

While a proteomics approach proved successful for identifying the animal hair fibers as coming from camelids, highly conserved peptide sequences make it difficult to parse out individual species within the genus of *Lama*. A recent study identified a few diagnostic peptides for separating between camelids, in particular for distinguishing the two wild species (guanaco and vicuña).^64^ One of these diagnostic peptides is a keratin K86 peptide that consistently appears for the domesticated llama and alpaca. This sequence (EYQEVLNSK) was recovered for the red fibers extracted from P.6862.1.5 and P.6862.1.9 and from a green fiber for P.6862.1.5. The red fibers from P.6862.1.5 and P.6862.1.9 also contained the sequence NSLENTLTETEAR, which was mentioned in the same study^64^ as another camelid marker usually absent in alpaca. For the yellow fiber from P.6862.1.3 and the green fiber from P.6862.1.5, this sequence had DSLENTLTETEAR, instead, which, according to the BLAST results, was a high match for *Vicugna pacos* or alpaca.

The appearance of these diagnostic peptides in these three fibers suggests that the state of preservation of the animal hair will dictate whether or not species identification via proteomics is possible. The high number of recovered peptides for the two red samples is consistent with prior work suggesting that certain anthraquinone dyes have antimicrobial effects.^65^ It is less clear why the green fiber from P.6862.1.5 yielded better results, though it is possible that the use of a particular mordant, like alum or copper, could have had similarly toxic effects that helped prevent biodegradation.^66^ Peru has natural sources of both alum salts and copper rich clays, making the use of these mordants likely.^67^

## Conclusions

Valuable information about the history of a piece, as well as its conservation state, can come through understanding the materials used to create it. The primary functions of identifying the raw materials in textiles are to understand the technical expertise of a particular civilization, to understand the potential availability and the exchange of natural resources (which can be used to place a material object in a particular time-period) and to plan both conservation and restoration efforts when necessary. From the working practices apparent in ancient textiles it is also possible to extrapolate to the cultural significance of certain artifacts and the role textiles might have played in social relations as a symbol of power.

The combined analytical approach taken here allowed for the identification of the natural dye extracts and the animal species used for Chancay textiles. From the identified dye marker compounds the textile fragments examined contained dyes of a range of chemical classes, including indigoid, anthraquinone and flavonoid. Comparison of three different extraction protocols showed that dye material was most efficiently extracted using the formic acid/methanol approach devised for flavonoid dyes, although good results could also be obtained for most dye classes using isopropanol and ammonium bicarbonate, two of the materials utilized during protein extraction via the direct digestion method. This suggests that direct digestion can be a simple, yet effective, means of extracting both dyes and protein. Simplifying the number of solvents and steps required for extracting either dyes or proteins has the added benefit of minimizing potential sample loss or contamination.

## Supporting Information Available

Identified dye marker compounds and proposed sources; and summary of the weave structure, colors, fiber composition and analytical techniques applied for the studied samples (PDF)
Dye marker compounds (XLSX)
Peptides detected for each sample (XLSX)
Species assignment based on BLAST results (XLSX)
