# Supplementary material for: Investigation of Natural Dyes and Taxonomic Identification of Fibers Used in Chancay Textiles by Vibrational Spectroscopy and Mass Spectrometry
Source: J Proteome Res. 2025 Jan 3;24(2):710–28. doi: 10.1021/acs.jproteome.4c00809 (PMC11812008; doi:10.1021/acs.jproteome.4c00809)
Supplement: Supplementary file 1 — pr4c00809_si_001.pdf [file pr4c00809_si_001.pdf]

Supplementary Information

Investigation of natural dyes and taxonomic identification of fibers used in Chancay textiles by vibrational spectroscopy and mass spectrometry

Katja S. Diaz-Granados[1]*, Laura J. Bergemann[2], Mary Ballard[3], G. Asher Newsome[3], Gwénaëlle M. Kavich[3], Joshua D. Caldwell[4], Timothy P. Cleland[3]

(1) Interdisciplinary Materials Science, Vanderbilt University, Nashville, Tennessee 37212, United States;

(2) The Conservation Center, Institute of Fine Arts, New York University, New York 10075 New York, United States;

(3) Museum Conservation Institute, Smithsonian Institution, Suitland 20746 Maryland, United States;

(4) Department of Mechanical Engineering, Vanderbilt University, Nashville, Tennessee 37212, United States;.

***Corresponding Author:** Katja S. Diaz-Granados[1], Interdisciplinary Materials Science, Vanderbilt University, Nashville, Tennessee 37212, United States; E-mail: katja.s.diaz-granados@vanderbilt.edu.

Table of Contents

Table S1. Dye marker compounds (XLSX)

Table S2. Identified dye marker compounds and proposed sources (PDF)

Table S3. Summary of the weave structure, colors, fiber composition and analytical techniques applied for the studied samples (PDF)

Table S4. Protein identification results (XLSX)

Table S5. Species assignment based on BLAST results (XLSX)

Table S2. Identified dye marker compounds and proposed sources.

**Fiber Sample**	**Marker Compounds**	**Dye Class**	**Source**
P.6862.1.1 Blue	indigotin, 2--indoline, caffeic acid, indoxyl acetate, isatin	indigoid	Indigofera, Eupatorium or Cybistax
P.6862.1.8 Blue	indigotin, 2--indoline, caffeic acid, indoxyl acetate, isatin, indican	indigoid	Indigofera, Eupatorium or Cybistax
P.6862.1.5 Red	carminic acid, dcII, laccaic acid D, kermesic acid, lucidin, rubiadin, alizarin, purpurin, pseudo-purpurin	anthraquinone	Dactylopius and Relbunium
P.6862.1.9 Red	carminic acid, dcII, laccaic acid D, kermesic acid, lucidin, rubiadin, alizarin, purpurin, pseudo-purpurin	anthraquinone	Dactylopius and Relbunium
P.6862.1.3 Yellow	hydroxybenzoic acids, tetrahydroxymethoxy-flavone, 5-O-caffeoylquinic acid, quercetin-4-glucoside, isorhamnetin-3-glucoside, trifolin, maclurin, dimethylquercetagetin and patuletin	flavonoid	Baccharis, Bidens, Hypercium
P.6862.1.4 Yellow	hydroxybenzoic acids, sideritiflavone, okanin,	flavonoid	Asteraceae

	butein, caffeic acid, quercetin and purpurin		
P.6862.1.5 Yellow	hydroxybenzoic acids, sideritiflavone,brazilin, okanin, purpurin, caffeinic acid, isorhamnetin-3-glucoside, trifolin, quercetin, butein, sulfuretin, marimetin, tetrahydroxymethoxy-flavone and gallic acid	flavonoid	Asteraceae
P.6862.1.6 Yellow	hydroxybenzoic acids, sideritiflavone, mangrovamide J, sulfuretin, maritimetin, quercetin-3-sulfate, caffeinic acid, caffeic acid, trifolin, quercetin-4-glucoside and purpurin	flavonoid	Cosmos or Bidens
P.6862.1.9 Yellow	hydroxybenzoic acids, caffeinic acid, caffeic acid, butein, gallic acid, di-O-caffeoylquinic acid and quercetin-4-glucoside	flavonoid	Asteraceae
P.6862.1.4 Green	indigotin, 3,4-dihydroxybenzoic acid,	flavonoid and indigoid	Asteraceae and Fabaceae

	isatin, indirubin, caffeic acid, brazilin, okanin, hematoxylin		
P.6862.1.5 Green	indigotin, sulfuretin, 3,4-dihydroxybenzoic acid, caffeic acid, indirubin, butein, brazilin	flavonoid and indigoid	Asteraceae and Fabaceae
P.6862.1.5 Black	carminic acid, dcII, dcofk, flavokermesic acid glucopyranoside, dcVII, laccaic acid D, hydroxybenzoic acid, emodin and caffeinic acid	flavonoid, anthraquinone and indigoid	Asteraceae, Fabaceae and Dactylopiidae
P.6862.1.9 Black	hydroxybenzoic acid, carminic acid, dcVII, butein, caffeinic acid, caffeic acid, luteolin dimethyl ether, luteolin methyl ether, dihydroxy-methyl-anthraquinone, methoxyanthraquinone, ellagic acid and indirubin	flavonoid, anthraquinone and indigoid	Asteraceae, Fabaceae and Dactylopiidae

Table S3. Summary of the weave structure, colors, fiber composition and analytical techniques applied for the studied samples.

Textile	Weave structure	Colors Studied	FT-IR	LC-MS	DART-MS
**P.6862.1.1** 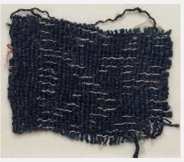	Supplementary weft weave (weft-patterned weave)	Blue	Cellulose Fiber		X
**P.6862.1.2** 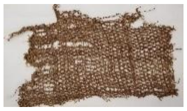	Split gauze/fancy gauze/Peruvian gauze	Undyed	Cellulose Fiber		
**P.6862.1.3** 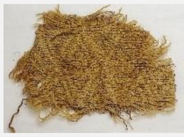	Supplementary weft weave (weft-patterned weave)	Yellow	Proteinaceous Fiber	X	X
**P.6862.1.4** 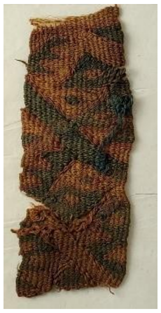	Slit Tapestry	Green	Cellulose Fiber	X	X
**P.6862.1.5** 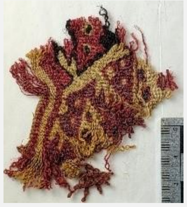	Slit Tapestry	Black, green, yellow, red	Proteinaceous Fiber	X	X
**P.6862.1.6**	Supplementary weft weave (weft-patterned weave)	Yellow	Proteinaceous Fiber (yellow); Cellulose Fiber (brown)	X	X

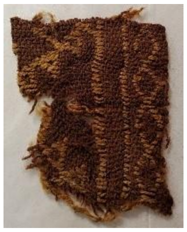					
**P.6862.1.7** 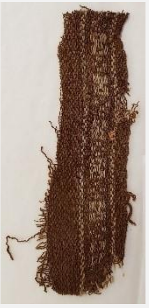	Extended plain weave or half basket weave (plain weave with paired warp or weft)	Undyed	Cellulose Fiber		
**P.6862.1.8** 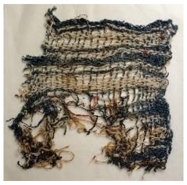	Plain weave	Red, blue	Proteinaceous Fiber (red)	X	X
**P.6862.1.9** 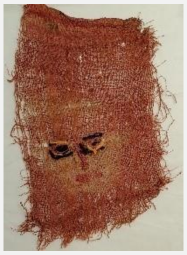	Plain weave with embroidery	Black, red, yellow	Proteinaceous Fiber (embroidery); Cellulose Fiber (background)	X	X
